# Key messengers in the gut-nose axis: mechanisms of gut microbial metabolites in the immunomodulation of allergic rhinitis

**DOI:** 10.3389/fimmu.2026.1796775

**Published:** 2026-04-17

**Authors:** Rong-Jing Qie, Jiang-Bo Qin, Hong-Yu Wu, Zhong-Hao Ji

**Affiliations:** 1 Department of Otorhinolaryngology-Head and Neck Surgery, Changzhi People’s Hospital Affiliated to Changzhi Medical College, Changzhi, Shanxi, China; 2 Beihua University School of Basic Medical Science, Beihua University, Jilin, China; 3 Laboratory of Brain Diseases and Cognitive Behavior, Department of Basic Medicine, Changzhi Medical College, Changzhi, Shanxi, China

**Keywords:** allergic rhinitis, bile acids, gut-nose axis, immunomodulation, polyamines, short-chain fatty acids, tryptophan metabolites

## Abstract

Allergic rhinitis (AR) is a common clinical chronic inflammatory respiratory disease, in which immune imbalance serves as a core component of its complex pathogenesis. In recent years, the gut-nose axis has emerged as a novel pathway mediating immune crosstalk between the intestinal tract and the nasal cavity, garnering significant academic attention. Gut microbial metabolites (such as short-chain fatty acids, tryptophan metabolites, bile acids, and polyamines) are profoundly involved in the pathophysiology of AR by reshaping the nasal mucosal immune microenvironment via systemic circulation and neural pathways and regulating the Th2/Treg balance, innate lymphoid cells (ILC2s), and mast cell functions. This article systematically reviews the immunomodulatory mechanisms of core gut microbial metabolites, explores their impact on nasal mucosal epithelial barrier function and immune cell activity, and summarizes metabolite-based clinical intervention strategies, including postbiotic therapy (bioactive compounds derived from microbial cells or metabolites), precision nutritional interventions, and fecal microbiota transplantation. Additionally, the paper analyzes current challenges such as heterogeneity and dose-response effects, aiming to provide a theoretical foundation for understanding the immunomodulatory mechanisms of the gut-nose axis and a reference for developing novel precision strategies for the prevention and treatment of AR.

## 1 Introduction

Allergic Rhinitis (AR) is a common clinical chronic inflammatory respiratory disease characterized by an aberrant immune response of the nasal mucosa to specific allergens. Its clinical symptoms, including nasal congestion, rhinorrhea, and sneezing, severely impair the quality of life of patients.

The role of gut microbiota and their metabolites in the immune evolution of AR has become increasingly prominent. Systematic reviews indicate that the structural composition of the gut microbiota undergoes characteristic changes in AR patients, suggesting that dysbiosis and its associated metabolic disturbances are deeply involved in the immune imbalance of AR (1). Gut microbial metabolites can directly regulate the activation of immune cells and the secretion of inflammatory cytokines (2). Moreover, impaired intestinal barrier function and the activation of related inflammatory signaling pathways can exacerbate nasal mucosal inflammation, forming a pathophysiological coupling within the gut-nose axis (3).

In recent years, with the deepening of research in immunology and microbiology, the gut-nose axis has emerged as a critical pathway connecting the intestinal microecology and the nasal immune system, becoming a prominent research hotspot. Gut microbiota and their metabolites significantly influence the inflammatory state and immune tolerance of the nasal mucosa by precisely modulating local and systemic immune responses, providing a vital direction for revealing new mechanisms of AR pathogenesis and developing precision therapeutic strategies. In-depth exploration of the role of gut metabolites in AR immunomodulation not only helps to resolve new mechanisms of disease onset but also provides potential therapeutic targets for clinical practice, promoting the development of personalized precision medicine.

## 2 Anatomical and physiological basis of the gut-nose axis

### 2.1 Definition and composition of the gut-nose axis

The gut-nose axis refers to the bidirectional physiological connection between the intestinal tract and the nasal cavity mediated by immune signals and neural pathways. This concept provides a novel perspective for understanding the immunomodulation of respiratory diseases such as AR. This axis involves a complex intertwining of the gut and nasal microbiota and their metabolites, forming a sophisticated regulatory network (3). The core mechanism of the gut-nose axis lies in the remote shaping of the nasal mucosal immune microenvironment by the intestinal microecology; conversely, local inflammatory states in the nasal cavity can provide feedback to regulate intestinal immune responses, thereby achieving a dynamic balance of systemic immune homeostasis.

The structural composition of the gut-nose axis primarily encompasses the gut microbiota, the gut mucosal immune system, the circulatory system, and the nasal mucosal immune environment. Gut microbes mediate signal transduction and immunomodulation across the gut-nose axis by producing bioactive metabolites such as short-chain fatty acids (SCFAs) and indole derivatives (4). The gut mucosal immune system participates in this axial linkage by regulating the activation states and directional migration of immune cells; for example, specific γδT17 cells can migrate along the “gut-lung-nose” axis to participate in distal inflammatory regulation (5). The circulatory system serves as a vehicle for substance transport and cellular migration, effectively connecting the gut and nasal cavity to ensure the efficient delivery of microbial metabolites and cytokines between both ends.

The nasal mucosal immune environment is composed of epithelial cells, innate immune cells, and adaptive immune cells, serving as the primary defensive front against exogenous allergens and for maintaining nasal health. Nasal mucosal epithelial cells act not only as a physical barrier but also maintain barrier integrity through structures such as tight junctions (TJs), the dysfunction of which is considered a key pathogenic mechanism of AR (6). Innate immune cells also play a central role in the nasal mucosa; studies have shown that nasal epithelial cells can activate innate immune responses through specific signal transduction pathways to counter pathogen invasion (7). Locally within the nasal cavity, macrophages and dendritic cells finely tune the immune response by recognizing and engulfing antigens and releasing cytokines.

### 2.2 Interaction between gut microbiota and host immunity

The gut microbiota is a complex and dynamic ecosystem within the human body, encompassing a wide variety of microorganisms such as bacteria, fungi, and viruses. It is deeply involved in regulating host physiological functions, playing a particularly critical role in the development and maintenance of immune system homeostasis. By producing bioactive molecules—such as SCFAs, secondary bile acids, and tryptophan metabolites—gut microbes remotely modulate local and systemic immune responses, thereby influencing the physiological state of distal organs. For instance, gut microbial metabolites participate in the progression of “gut-lung axis” related diseases by regulating respiratory immunity (8); simultaneously, the interaction between the microbiota and bile acids occupies a central position in the immune and metabolic regulation of the “gut-liver axis” (9). This cross-organ immune communication network highlights the status of the gut microbiota as a central hub for the body’s overall immune regulation.

The interaction between gut microbiota and the host immune system is bidirectional and dynamic. The body’s innate and adaptive immune cells maintain the stability of microbial community structure by finely regulating the biogeographical distribution of gut microbes (10). Various immune cells, including dendritic cells, macrophages, B cells, and T cells, are all key factors in maintaining the balance of the intestinal microecology. When the immune system is impaired or deficient, the diversity and abundance of gut microbes undergo significant shifts, a phenomenon that is particularly prominent in the small intestinal mucosa (10). The stability of this “immune-microbe” reciprocal relationship is essential for the host to resist pathogen invasion, induce immune tolerance, and prevent immunopathological damage.

### 2.3 Immune structure and function of the nasal mucosa

As the primary barrier of respiratory defense, the nasal mucosa possesses a complex immune architecture designed to efficiently resist exogenous antigen invasion and maintain local homeostasis. The nasal mucosa is enriched with various immune cells, including dendritic cells (DCs), mast cells, and type 2 innate lymphoid cells (ILC2s), which play central roles in local immune surveillance and response. Single-cell RNA sequencing studies have revealed that the proportion of innate immune cells in the nasal mucosa undergoes dynamic adjustments with age, suggesting that the colonization and composition of immune cells are of great significance for the precise regulation of immune responses (11). As professional antigen-presenting cells, DCs are responsible for capturing and processing inhaled allergens, thereby initiating local and systemic immune responses. Mast cells are localized in the superficial layer of the mucosa and serve as the core effector cells of allergic reactions; their IgE-dependent activation is a key link in the pathological process of allergic rhinitis (12). As resident immune populations, ILC2s are active during both short-term and long-term antigen stimulation, acting as a bridge between innate and adaptive immunity and mediating the onset and persistence of inflammation (13).

The barrier function of the nasal mucosa is also a core element in maintaining homeostasis. The epithelial layer constructs a chemical barrier by secreting antimicrobial peptides (such as β-defensin 2) and various cytokines, including IL-4, IL-6, IL-10, and IL-12, and is deeply involved in the regulation of immune responses and cell differentiation processes (14). Meanwhile, the mucus layer and ciliary movement work synergistically to clear non-specific particles and pathogens, maintaining the cleanliness and functional stability of the respiratory tract (15). Nasal-associated lymphoid tissue, serving as a site for local immune induction, exerts a mucosal protective effect by producing secretory IgA (16). Clinical and basic research further confirms that the nasal mucosal immune response is closely related to the systemic immune state: the expression levels of specific immune genes in the nasal mucosa are positively correlated with peripheral blood eosinophil counts and specific IgE levels, reflecting the deep interaction between local and systemic immunity (17). Additionally, studies have found that SARS-CoV-2 mRNA vaccination can also induce significant changes in nasal mucosal antibody levels and microbiota composition (18, 19).

### 2.4 Signal transduction mechanisms of the gut-nose axis

Gut microbial metabolites reshape the immune microenvironment of the nasal mucosa through multiple pathways, the core mechanisms of which primarily include systemic circulatory conduction and neural signaling regulation. Bioactive molecules produced by the gut microbiota—such as SCFAs, tryptophan metabolites, and aromatic amino acid derivatives—can cross the intestinal barrier into the blood circulation to achieve remote modulation of immune cell functions and inflammatory responses in the nasal mucosa. For example, gut-derived tryptophan metabolites significantly improve intestinal barrier function and enhance anti-inflammatory effects by activating the aryl hydrocarbon receptor (AhR) signaling pathway (20), which in turn reduces systemic pro-inflammatory signals and suppresses the hyperresponsiveness of the nasal mucosa; meanwhile, the metabolite phenylacetylglutamine has been confirmed to exert systemic regulatory effects by activating adrenergic receptors (21). These metabolites serve as key messenger molecules, acting as a bridge in coordinating immune homeostasis between the gut and the nasal cavity.

The directed migration of immune cells is another critical link in the signal transduction of the gut-nose axis. Research has found that gut-derived γδT17 cells can shuttle and migrate among the gut, lung, and nasal mucosa via a specific pathway mediated by integrin α4β7, influencing the immune-inflammatory state of the respiratory tract by regulating local IL-17A levels. The Traditional Chinese Medicine formula Ma-Xing-Shi-Gan-Tang has been shown to reduce the chemotactic recruitment of γδT17 cells to the nose by inhibiting the CCL25/α4β7 axis, thereby alleviating nasal inflammation associated with “Lung-Heat syndrome.” This reveals the regulatory mechanism of immune migration along the “gut-lung-nose” axis at a molecular level (5). This finding highlights the vital role of immune cell trafficking in gut-derived signal regulation of nasal immunity, suggesting that intervening in the expression of relevant chemokines and integrins is a potential new strategy for treating AR.

Furthermore, the dynamic restructuring of the cytokine network is a key mechanism in gut-nose axis regulation. Gut microbes and their metabolites can modulate the expression of various core inflammatory factors, including IL-4, IL-10, IFN-γ, and IL-17A. Fecal microbiota transplantation (FMT) studies have demonstrated that restoring gut microbiota balance can significantly attenuate nasal mucosal inflammation in AR mouse models. This mechanism involves the precise regulation of Th1/Th2/Th17-related transcription factors and cytokines, as well as the effective promotion of epithelial barrier protein expression (22). Additionally, Bavachinin repairs the nasal mucosal barrier and improves the intestinal microecology by inhibiting NLRP3 inflammasome-mediated cell pyroptosis via the PI3K/AKT/NF-κB signaling pathway (23).

### 2.5 Evidence for the role of the gut-nose axis in allergic rhinitis

In recent years, both clinical studies and animal experiments have confirmed that gut microbiota dysbiosis is significantly correlated with the severity of AR. A 16S rRNA gene sequencing study involving 23 AR patients and 15 healthy controls revealed a significant shift in the gut microbiota structure of AR patients, specifically characterized by a decrease in the abundance of beneficial SCFA-producing bacteria (such as *Faecalibacterium*) and an abnormal enrichment of pro-inflammatory bacteria (such as *Fusobacterium*). This phenomenon suggests a close association between gut microecological imbalance and the pathogenesis of AR (24). Furthermore, studies on AR mouse models have further revealed the synchronized evolution of gut and nasal microbiota and their interaction with metabolites, where the bidirectional regulatory effects of this gut-nose axis collectively exacerbate nasal mucosal inflammation and tissue damage (3).

Intestinal metabolic disturbances are highly correlated with the inflammatory state of the nasal mucosa. Specifically, microbial metabolites such as indole-3-propionic acid and succinate have been confirmed to be closely related to the gene expression levels of key AR inflammatory mediators (e.g., IL-6, TNF, and IL-1β) (25). Serving as key molecular bridges of the gut-nose axis, these metabolites are deeply involved in the regulation of the nasal mucosal immune response by modulating inflammatory signaling pathways and immune cell functions. Additionally, the downregulation of SCFA levels mediated by dysbiosis impairs intestinal barrier function and disrupts immune homeostasis, thereby inducing a systemic inflammatory state and ultimately enhancing nasal mucosal hypersensitivity (26).

Animal experiments have further established the causal link between gut microbiota and nasal mucosal inflammation. Restoring the gut microbiota structure in AR mice through FMT can significantly alleviate nasal symptoms and inflammatory responses. The underlying mechanisms involve the upregulation of tight junction proteins (such as ZO-1 and claudin-1) in both the gut and nasal mucosa, the regulation of CD4+ T-cell subset balance, and the inhibition of the overactivation of the PI3K/AKT/mTOR and NF-κB signaling pathways (22). These results indicate that the recovery of gut microbiota not only optimizes the local intestinal microenvironment but also mitigates allergic inflammation in the distal nasal cavity through systemic immune regulation, strongly supporting the central role of the gut-nose axis in the pathogenesis of AR.

In summary, multi-dimensional evidence from clinical and animal models consistently demonstrates that the gut-nose axis plays a pivotal regulatory role in the occurrence and development of allergic rhinitis. Gut microbiota dysbiosis and alterations in the metabolite profile modulate the nasal mucosal immune response through multi-level signaling pathways.

## 3 Core metabolites and their regulatory mechanisms

A variety of key gut microbiota-derived metabolites, such as SCFAs, tryptophan metabolites, bile acids, and polyamines, serve as core signaling molecules within the gut-nose axis, synergistically exerting immunomodulatory and barrier-protective effects (**Table 1**). While certain metabolite-mediated gut-organ communication mechanisms were initially identified in the gut-lung or gut-heart axes, their analogous expression in the nasal mucosa suggests a potential cross-organ consistency; however, this remains to be further validated in AR models.

**Table 1:** Core metabolites and their regulatory mechanisms.

Metabolite type	Key members	Sources	Key receptors/pathways	Regulatory mechanism	References
SCFAs	Acetic acid, Propionic acid, Butyric acid	*Faecalibacterium*,*Roseburia*	GPCRs and HDAC inhibition	Modulating the Treg/Th2 balance; Enhancing barrier function; Regulating cytokine expression; Inhibiting Th2 immune polarization.	(27–36)
Tryptophan metabolites	ILA, IPA, IAA, 3-IAld	*Lactobacillus*,*Bifidobacterium*	AhR, Nrf2, STAT3	Barrier repair; Gut-lung axis regulation; Maintenance of immune homeostasis.	(37–42)
Bile acids (secondary bile acids)	DCA, LCA, THDCA	*Clostridium*,*Bacteroides*	FXR,TGR5	Induce anti-inflammatory polarization; Mediate antimicrobial peptide expression; Regulate cytokine balance; Alleviate cellular stress.	(43–46)
Polyamines	Putrescine, spermine, and spermidine	Microbial metabolism of proteins and amino acids	Polyamine transporter, autophagy pathway	Enhancing Treg-mediated immunomodulation; Inducing M2 macrophage polarization; Promoting cell proliferation and differentiation.	(47–49)
Lipids/ketone bodies	β-Hydroxybutyrate (BHB), conjugated linoleic acid (CLA), lipid mediators	Metabolites/Ketogenesis	GPR109A,STAT1/4 ,CRTH2	Modulating the ILC2-mast cell axis; Promoting epithelial repair; Regulating allergic inflammatory mediators.	(50–54)

### 3.1 Short-chain fatty acids

SCFAs, primarily comprising acetate, propionate, and butyrate, are the major metabolites produced by the fermentation of dietary fiber by gut commensal bacteria. SCFAs serve not only as energy substrates for the host but also participate deeply in the regulation of immune system functions through various mechanisms, acting as “immune brakes” to maintain immune homeostasis and effectively inhibit excessive inflammatory responses (36).

Firstly, SCFAs modulate immune cell activity by activating specific G-protein-coupled receptors (GPCRs), such as GPR41 (FFAR3) and GPR43 (FFAR2). These receptors are widely expressed on the surfaces of Tregs, DCs, macrophages, and neutrophils. Studies have confirmed that the binding of SCFAs to GPR43 promotes the differentiation and functional enhancement of Treg cells, thereby increasing their capacity to suppress Th2-mediated allergic inflammation and alleviating immune damage in allergic diseases such as AR (35). Additionally, SCFAs can regulate gene expression and immune cell phenotypic transformation at the epigenetic level by inhibiting histone deacetylase (HDAC) activity, further inducing immune tolerance and anti-inflammatory responses (34).

Secondly, SCFAs protect nasal tissues from allergic inflammation by strengthening nasal mucosal barrier function and reducing the release of inflammatory cytokines. Specific mechanisms include upregulating the expression of tight junction proteins (such as ZO-1 and Occludin) to maintain the integrity of the mucosal epithelial barrier and block the penetration of allergens and inflammatory mediators (32, 33). Among them, butyrate is considered a critical metabolite for maintaining both intestinal and distal mucosal barriers, butyrate inhibits HDAC activity to promote acetylation of the Foxp3 gene promoter, thereby enhancing Treg cell homeostasis and further inducing immune tolerance and anti-inflammatory responses (33).

Moreover, SCFAs are capable of reshaping the inflammation-related cytokine network. By decreasing the levels of Th2-type cytokines such as IL-4 and IL-5 while increasing the expression of the anti-inflammatory factor IL-10, SCFAs effectively correct the imbalance in Th1/Th2 and Treg/Th17 cell ratios, thereby improving the state of allergic inflammation (29, 33). This immunomodulatory effect is not limited to the local intestine but extends to the nasal immune microenvironment via the gut-nose axis, highlighting the central role of gut microbial metabolites in distal mucosal immune regulation.

In summary, SCFAs produced by gut microbes act as key signaling molecules that promote Treg cell differentiation, inhibit Th2-mediated allergic responses, and enhance nasal mucosal barrier function through multiple pathways, including the activation of GPCRs and the inhibition of HDAC activity.

### 3.2 Tryptophan metabolites

Tryptophan and its metabolites play a central role in maintaining intestinal barrier integrity and inducing immune tolerance. The gut microbiota metabolizes tryptophan into various indole derivatives, such as indole-3-lactic acid (ILA), indole-3-propionic acid (IPA), indole-3-acetic acid (IAA), and indole-3-carboxaldehyde (3-IAld). These molecules serve as endogenous ligands for the AhR, promoting epithelial cell repair, enhancing the expression of tight junction proteins, and inducing mucus layer formation, thereby significantly strengthening intestinal barrier function. For example, ILA produced by Lactiplantibacillus plantarum DPUL-S164 activates the AhR/Nrf2 signaling pathway to upregulate tight junction protein levels, effectively alleviating intestinal inflammation and barrier damage (41, 42). IPA has been confirmed to increase transepithelial electrical resistance, reinforce the protective effect of the mucus layer, and inhibit the expression of pro-inflammatory cytokines, further consolidating intestinal homeostasis (40).

Tryptophan metabolites suppress allergic inflammation by finely regulating immune cell functions. Research has shown that gut-derived tryptophan metabolites mediate the activation and migration of ILC3s through the AhR/STAT3/IL-22 pathway, enhancing respiratory barrier function and mitigating infection-related inflammation (39). Concurrently, these metabolites can inhibit excessive degranulation of mast cells and reduce the release of histamine and inflammatory mediators, thereby alleviating the clinical symptoms of AR. Furthermore, AhR activation plays a key role in maintaining the Th17/Treg cell balance. Numerous studies indicate that tryptophan metabolic defects or impaired AhR signaling lead to mucosal barrier breakdown and immune imbalance, increasing susceptibility to allergic and inflammatory diseases (37, 38).

In summary, as vital messengers of the gut-nose axis, tryptophan metabolites promote epithelial repair, reinforce barrier integrity, and regulate ILC2s, mast cells, and the Th17/Treg balance by activating the AhR signaling pathway, thereby exerting multi-dimensional immunomodulatory effects.

### 3.3 Bile acids and polyamines: potential immunomodulatory factors

Bile acids (BAs), as core products of gut microbial metabolism, exert precise immunomodulatory effects by binding to host-specific receptors—the farnesoid X receptor (FXR) and the G protein-coupled receptor TGR5 (also known as GPBAR1). FXR is primarily expressed in the liver and intestinal epithelial cells; while maintaining bile acid circulation, it can inhibit the release of pro-inflammatory cytokines and alleviate systemic inflammation. TGR5 is widely distributed on immune cells, and its activation can promote the secretion of anti-inflammatory factors and regulate the functions of macrophages and DCs. Gut microbiota convert primary bile acids into various secondary bile acids, which serve as potent endogenous ligands for FXR and TGR5, not only regulating intestinal homeostasis but also influencing inflammatory responses in distal tissues (46). Research indicates that bile acids, by activating these receptors, can induce macrophage polarization toward the M2 anti-inflammatory phenotype and inhibit the release of inflammatory mediators (55). Simultaneously, bile acids act as a bridge between innate and adaptive immunity by regulating DC antigen presentation and T-cell activation (46). In the pathophysiology of AR, bile acid-mediated signaling pathways may participate in reshaping the local immune microenvironment of the nasal mucosa, thereby mitigating inflammation-induced tissue damage.

Polyamines, including putrescine, spermine, and spermidine, are important bioactive products of gut microbial metabolism of proteins and amino acids, and they are widely involved in cell proliferation, differentiation, and immune regulation. Polyamines are not only essential factors for cell growth but also enhance the activity of Tregs and inhibit the production of pro-inflammatory cytokines, thereby exerting significant anti-inflammatory effects (48, 49). Furthermore, polyamines can induce M2 macrophage polarization, enhance tissue repair capacity, and finely regulate antigen presentation by DCs to maintain adaptive immune balance (47). These properties make polyamines key effector molecules in the gut-nose axis for regulating nasal mucosal immune homeostasis, possessing potential value for alleviating the inflammatory symptoms of AR.

In summary, as key metabolic messengers in the gut-nose axis, bile acids and polyamines regulate local nasal mucosal immune responses through receptor-mediated signaling cascades and promote the establishment of immune tolerance.

## 4 Remodeling of specific cell subsets in the nasal mucosa by metabolites

**Figure 1:**
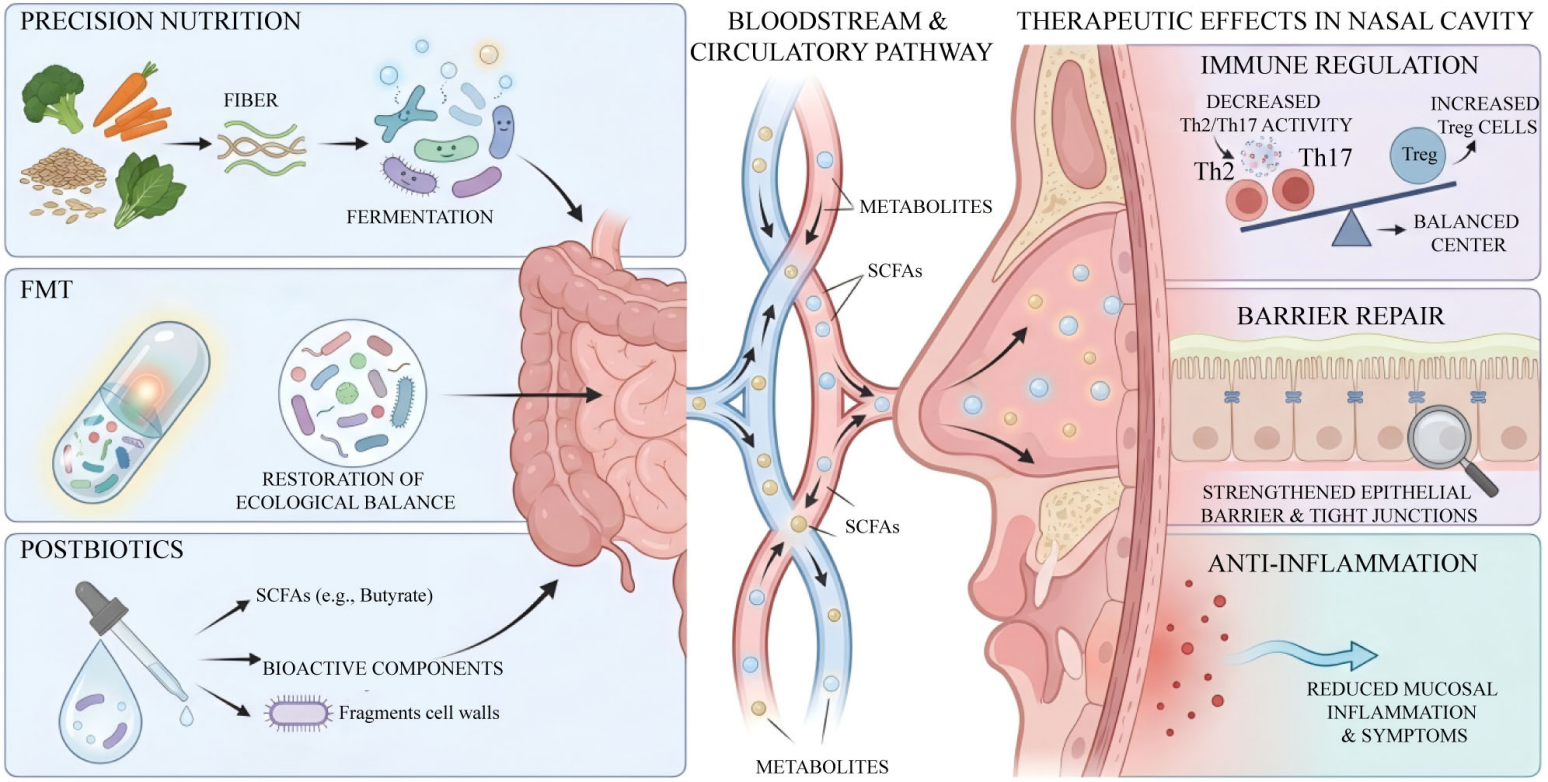
Schematic diagram of the mechanisms by which gut microbial metabolites regulate nasal mucosal immune homeostasis and barrier function. Gut microbial metabolites act on the nasal cavity through the circulatory system and neural pathways. By modulating immune balance and fortifying epithelial barrier functions, these metabolites synergistically maintain the homeostasis of the nasal microenvironment.

Gut microbial metabolites exert pivotal anti-inflammatory and reparative effects within the gut-nose axis through multi-dimensional immunomodulatory mechanisms, thereby effectively alleviating AR (**Figure 1**).

### 4.1 Epithelial cells

Gut microbial metabolites play a central role in maintaining epithelial barrier function, particularly showing significant efficacy in promoting the expression of tight junction proteins such as ZO-1, Claudin-1, and Occludin. Studies have confirmed that SCFAs produced by the gut microbiota can activate G-protein-coupled receptors (e.g., GPR41, GPR43) on the surface of epithelial cells and inhibit HDAC activity, thereby upregulating tight junction protein expression and enhancing barrier integrity (31). Furthermore, probiotics and their metabolites precisely regulate epithelial gene expression to promote barrier protein synthesis, significantly reducing mucosal permeability and enhancing barrier stability (56). This regulatory effect is not limited to the local intestine but also extends to distal respiratory epithelia, such as the nasal mucosa, via the gut-nose axis, reflecting the universality of microbial metabolite-mediated cross-organ regulation of barrier function.

Regarding the mechanisms of cell apoptosis and repair, microbial metabolites also possess key regulatory functions. SCFAs, represented by butyrate, can effectively inhibit epithelial cell apoptosis and promote cell proliferation and tissue repair by downregulating inflammatory cytokine levels and optimizing cellular energy metabolism (30). Additionally, conjugated linoleic acid (CLA) metabolized by the gut microbiota activates the STAT1/4 signaling pathway to induce macrophage secretion of the anti-inflammatory factor IL-35, thereby indirectly promoting epithelial repair and maintaining immune homeostasis (53). The synergistic action of these mechanisms significantly enhances the overall homeostasis of the epithelial barrier and reduces the risk of mucosal damage.

Gut microbial metabolites can also alleviate mucosal injury by inhibiting the release of inflammatory mediators. Research has found that secondary bile acids promote the expression of the antimicrobial peptide Cathelicidin by activating the TGR5 receptor and its downstream ERK1/2 signaling pathway, enhancing epithelial antimicrobial defense while suppressing local inflammatory responses (45). Simultaneously, SCFAs alleviate mucosal inflammation by modulating the cytokine secretion profile of immune cells and reducing the levels of Th2-type pro-inflammatory cytokines such as IL-4, IL-5, and IL-13 (31). Furthermore, these metabolites can regulate endoplasmic reticulum stress responses in epithelial cells, alleviating the excessive release of inflammatory mediators and protecting the epithelial barrier from persistent erosion by chronic inflammation (43).

In summary, gut microbial metabolites significantly enhance the nasal mucosal barrier function in patients with allergic rhinitis through multiple pathways, including promoting barrier protein expression, regulating cell apoptosis and repair, and inhibiting the release of inflammatory mediators.

### 4.2 Th2/Treg balance

At the level of immunomodulatory mechanisms, programmed cell death protein 1 (PD-1) and its ligand (PD-L1) exhibit high expression levels in the nasal mucosa and peripheral blood of AR patients, showing a significant positive correlation with Th2 cell proportions and IgE levels. This suggests that the PD-1/PD-L1 signaling pathway may mediate the inflammatory process of AR by enhancing Th2-type immune responses (57). Furthermore, the CCL2/CCR2 chemokine axis is upregulated in AR rat models, driving inflammatory cell infiltration and the release of inflammatory mediators; studies have found that Evodiamine can improve AR immune function by inhibiting this pathway, highlighting the value of modulating chemokine signaling as a potential therapeutic target (58). Regulatory T cells (Tregs) and their epigenetic modifications play a central role in maintaining systemic immune homeostasis, and their functional impairment is closely associated with allergic diseases such as AR (59). Gut microbial metabolites play a pivotal role in maintaining the balance of helper T cell subsets, particularly in suppressing Th2 immune deviation and promoting the expansion of regs. Th2 cells are the core effector cells in allergic diseases such as AR; their overactivation mediates the secretion of cytokines like IL-4, IL-5, and IL-13, which in turn induces IgE synthesis and eosinophil recruitment, thereby exacerbating local inflammatory responses (60). Multiple studies have demonstrated that gut metabolites, such as SCFAs, can effectively inhibit the abnormal activation of Th2 cells and the release of their associated factors by intervening in immune cell metabolism and signaling pathways, thus correcting the Th2 immune bias (27, 28).

Tregs, as a critical component of immune regulation, limit excessive immune responses and prevent tissue damage by secreting inhibitory cytokines such as IL-10 and TGF-β (61, 62). The activation of the Notch2 signaling pathway is of great significance in Treg differentiation, as it promotes the differentiation of the GATA3+ Treg subset and enhances immunosuppressive efficacy to alleviate AR inflammation (63). Clinical studies have found that the decrease in the proportion of Tregs in the peripheral blood of AR patients is significantly negatively correlated with disease severity; meanwhile, gut microbial metabolites can induce the expansion and functional recovery of Foxp3+ Tregs, thereby reconstructing the state of systemic immune tolerance (64–66). These lines of evidence suggest that gut microbial metabolites provide an essential safeguard for alleviating AR-related immunoinflammation by precisely regulating the differentiation direction of T cell subsets.

The cytokine network serves as a crucial bridge for regulating the Th2/Treg balance. Th2-type factors such as IL-4 and IL-5 drive allergic reactions, while Treg-type factors such as IL-10 and TGF-β exert immunosuppressive effects to maintain systemic homeostasis (60, 64). Gut metabolites can reshape this cytokine network by lowering pro-inflammatory cytokine levels and increasing the secretion of anti-inflammatory factors, thereby alleviating allergic symptoms (66). For instance, taurohyodeoxycholic acid (THDCA), a gut metabolite, significantly downregulates IL-4 and upregulates the expression of IL-10 and TGF-β, correcting imbalances in Th1/Th2 and Th17/Treg ratios (44). Additionally, Tregs can limit the synergistic pro-inflammatory activity of Th2 cells via inhibitory factors, and the dynamic regulation of this network by metabolites constitutes a core link in the gut-nose axis”immune regulation (67, 68).

In summary, gut microbial metabolites play a vital regulatory role in alleviating the immunopathology of allergic rhinitis by regulating Th cell subsets through multiple targets, suppressing Th2 immune deviation, promoting the expansion and functional enhancement of Tregs, and maintaining immune tolerance through the restructuring of the cytokine network.

### 4.3 ILC2s and mast cells

ILC2s and mast cells, as key effector cells in AR, play a central role in the progression of local inflammatory responses and allergic symptoms. Research indicates that gut microbiota metabolites regulate ILC2 activation and cytokine secretion through multidimensional pathways, thereby influencing mast cell function and constructing a complex immunoregulatory network. First, metabolites such as short-chain fatty acids can directly or indirectly modulate ILC2 activity. For example, the key ketone body metabolite β-hydroxybutyrate (BHB) can inhibit mast cell function by activating the GPR109A receptor, blocking the IL-2-driven proliferation of ILC2s, thereby suppressing type 2 immune responses and the production of associated cytokines (54).

There is a significant reciprocal pro-inflammatory mechanism between ILC2s and mast cells. Type 2 cytokines (e.g., IL-5, IL-13) secreted by ILC2s promote the recruitment and activation of mast cells, while ILC2 activity itself is co-regulated by epithelial-derived alarmins (e.g., IL-33, TSLP, IL-25) and mast cell-derived lipid mediators (e.g., PGD2, CysLTs) (69, 70). These mast cell-derived mediators create a feedback loop that further amplifies ILC2-driven type 2 inflammation in AR. Specifically, PGD2 mediates the activation and chemotaxis of ILC2s through its receptor CRTH2, enhancing type 2 inflammation; studies have confirmed that blocking CRTH2 significantly alleviates airway hyperresponsiveness and inflammatory cell infiltration, suggesting that mast cell metabolites exert a crucial positive regulatory effect on ILC2 function (50). Additionally, certain metabolites effectively alleviate AR clinical symptoms by inhibiting the mast cell degranulation process and reducing the release of allergic mediators such as histamine (51, 52).

The remodeling of the local inflammatory microenvironment is another key link in regulating the functions of ILC2s and mast cells. By secreting IL-4, IL-5, IL-9, and IL-13, ILC2s induce eosinophil recruitment and reinforce mast cell effector functions, ultimately leading to mucus hypersecretion and airway remodeling (71, 72). Simultaneously, cytokines such as IL-18 can also promote the proliferation and activation of ILC2s, further amplifying allergic inflammation (73). Gut microbiota metabolites exert anti-inflammatory effects by intervening in the expression profiles of these cytokines, thereby indirectly modulating the local immune environment.

In summary, gut microbiota metabolites inhibit ILC2 activation and cytokine secretion through multi-level and multi-pathway mechanisms, while synergistically suppressing mast cell degranulation and histamine release.

## 5 Clinical translation and therapeutic strategies

**Figure 2:**
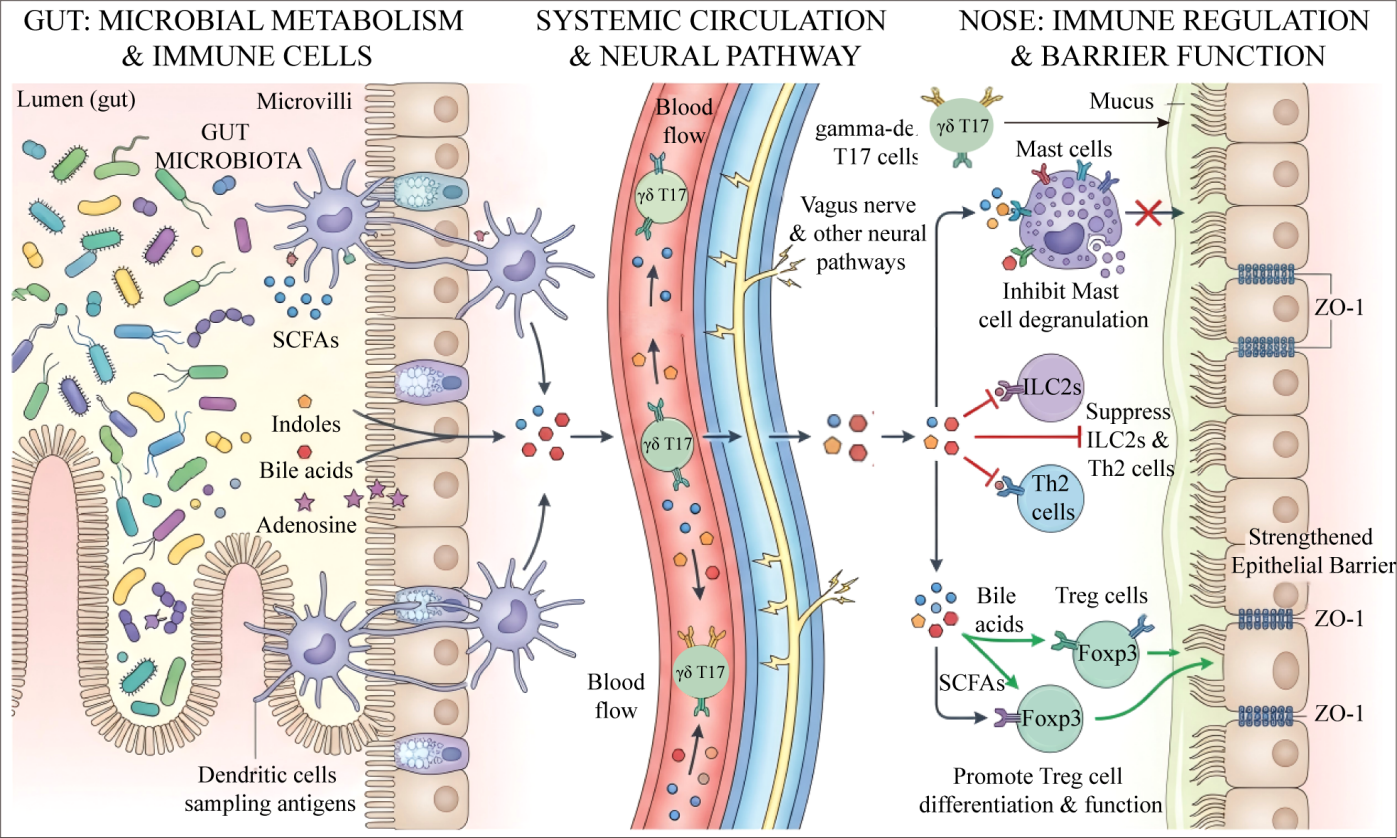
Schematic diagram of the mechanisms of gut microbiota intervention in nasal health based on the gut-nose axis. Intervening in the gut microbiota through approaches such as precision nutrition, FMT, and postbiotics promotes the delivery of metabolites like SCFAs via systemic circulation to target nasal tissues. This systemically improves nasal health through immunomodulation, barrier repair, and anti-inflammatory effects.

As the central role of the gut-nose axis in the pathogenesis of AR becomes increasingly prominent, intervention strategies targeting this axis have shifted from theoretical exploration to clinical translation. Gut microbial metabolites serve as key messengers mediating long-distance communication between the gut and the nasal cavity; an imbalance in their levels is a direct link to host immune dysregulation. Consequently, current clinical intervention logic has constructed a progressive framework—moving from the surface to the core, from “supplementing metabolic messengers” to “remodeling the micro-ecosystem”—aiming to provide a clear path for the precision treatment of AR through the systemic integration of different intervention dimensions (**Figure 2**).

### 5.1 Precision nutritional intervention

The gut microbiota also serves as a key hub between dietary nutrients and host immune responses (74, 75). Micronutrients are involved in regulating immune responses and maintaining the integrity of the mucosal barrier; their deficiency can significantly aggravate AR symptoms (76). Traditional Chinese medicines, such as Aconite (Fuzi), Dendrobium (Shihu), and their active components, demonstrate unique therapeutic advantages through anti-inflammatory and immunomodulatory effects, with the mechanism of Dendrobium being closely linked to the regulation of gut microbiota and related metabolic pathways (77, 78). Precision nutritional intervention aims to promote the production of beneficial gut metabolites through targeted dietary adjustments and has become an important strategy for modulating AR immune responses. The gut microbiome directly influences the functional state of the host immune system by producing metabolites such as SCFAs, vitamins, and polyamines. Changes in dietary structure can significantly reshape microbial abundance and metabolic activity, thereby indirectly regulating immune responses. For instance, increasing dietary fiber intake can specifically promote the expansion of probiotics and boost SCFA production, thereby strengthening intestinal barrier function and inducing immune tolerance (79). Furthermore, personalized dietary plans developed based on individual microbiome characteristics can maximize the biological effects of beneficial metabolites and enhance clinical therapeutic efficiency. This microbiome-based nutritional regulation strategy is not only applicable to the relief of allergic diseases but also provides new ideas for the prevention and treatment of other immune-related disorders.

A high-fiber diet is an effective way to induce SCFA production and plays a significant role in improving immune homeostasis. Fermentable fibers in the diet are degraded by the gut microbiota to produce SCFAs such as acetate, propionate, and butyrate. These metabolites exert immunomodulatory functions through various mechanisms, including activating Tregs, inhibiting the release of pro-inflammatory mediators, and maintaining the integrity of the mucosal barrier. Research indicates that a high-fiber diet can significantly reduce systemic inflammation levels and improve clinical symptoms in AR patients. SCFAs precisely regulate the differentiation and function of immune cells by binding to GPCRs on the surface of immune cells, promoting the reconstruction of immune homeostasis (80). Therefore, high-fiber dietary intervention is not only the cornerstone of regulating gut micro-ecology but also a core component of precision nutrition protocols.

Individualized nutritional plans based on microbiome characteristics can significantly optimize the therapeutic outcomes of AR. Given the significant individual differences in gut microbiota composition, a single dietary intervention model is unlikely to produce consistent efficacy across all patients. By deeply analyzing an individual’s microbial structure and metabolic potential, precision-designed nutritional plans—including adjusting fiber components, supplementing specific prebiotics or probiotics, and incorporating strategies such as time-restricted feeding—can achieve precise regulation of immune function (81). Furthermore, integrating metabolomics with immune phenotyping can further elucidate the interactive mechanisms between metabolites and host immune responses. Such protocols are expected not only to improve clinical efficacy and reduce side effects but also to provide a scientific basis for patients’ long-term health management.

### 5.2 Fecal microbiota transplantation

FMT restores microecological homeostasis by reshaping the structure of the gut microbiota, showing broad prospects for clinical translation in the treatment of various immune-related diseases in recent years. Gut dysbiosis is one of the core pathogenic factors in allergic diseases such as AR. By optimizing the diversity and composition of the gut microbiota, FMT promotes the colonization of beneficial bacteria and corrects metabolic disorders, thereby achieving systematic regulation of the host’s immune function. Studies have confirmed that FMT can repair the intestinal barrier and upregulate the expression of tight junction proteins such as ZO-1 and Claudin-1, maintaining intestinal mucosal integrity while reducing systemic inflammatory responses, thus alleviating allergic symptoms in the nasal mucosa (22). Furthermore, FMT effectively modulates the immune balance of CD4+ T cell subsets, exerting significant anti-inflammatory effects by correcting the imbalance of Th1/Th2/Th17 ratios.

Animal model studies have provided a solid experimental basis for the application of FMT in allergic diseases. In ovalbumin (OVA)-induced AR mouse models, FMT significantly alleviated nasal symptoms and mucosal pathological damage, reconstructed the gut microbiota structure, and mediated the recovery of immune homeostasis by inhibiting the activation of signaling pathways such as PI3K/AKT/mTOR and NF-κB (22). Clinical observations have also noted that some patients with ulcerative colitis experienced simultaneous improvement in their accompanying allergic rhinitis symptoms after receiving FMT, suggesting that the immunomodulatory effects of FMT possess systemic impacts that extend beyond the gastrointestinal tract (82). These lines of evidence collectively support FMT as an innovative strategy for regulating the immune function of the gut-nose axis. Although animal models have demonstrated the causal relationship of the gut-nose axis, differences in gut microbiota composition and nasal anatomy between rodents and humans limit the direct translation of these findings, highlighting the need for more multicenter human studies in the future.

Despite its enormous potential, the clinical promotion of FMT still faces challenges such as the need for standardized operating procedures and long-term safety assessments. Currently, a unified industry standard has not yet been reached regarding the implementation process, donor screening criteria, transplantation dosage, and frequency of FMT, and its long-term impact on the host immune system requires further in-depth investigation. Therefore, future research should focus on optimizing standardized operating procedures, establishing a comprehensive safety monitoring system, and verifying long-term efficacy through large-scale randomized controlled clinical trials. Additionally, integrating metabolomic characteristics to further resolve the molecular mechanisms by which FMT regulates immunity will help drive the formulation of individualized precision treatment protocols.

### 5.3 Postbiotic therapy

Postbiotics refer to bioactive substances derived from probiotics, including metabolites and structural components such as short-chain fatty acids, polypeptides, polysaccharides, and bacterial cell wall fragments. These components exert health-promoting effects without the requirement for viable microorganisms. Compared to probiotics, postbiotics offer superior physicochemical stability and biological safety. Since they do not contain live bacteria, they circumvent potential infection risks, making them particularly suitable for immunocompromised individuals or those sensitive to live microbial preparations (83). Furthermore, postbiotics possess clear immunomodulatory mechanisms, maintaining host immune homeostasis by suppressing inflammatory responses, strengthening epithelial barrier function, and precisely regulating immune cell activity, thereby demonstrating significant therapeutic potential.

Clinical studies indicate that postbiotics hold promising application prospects in the immune regulation of respiratory diseases, especially AR (56). Postbiotics effectively alleviate nasal mucosal inflammation and improve AR clinical symptoms by reinforcing the respiratory epithelial barrier, correcting the Th1/Th2 balance shift, and promoting the secretion of anti-inflammatory cytokines (84). Additionally, postbiotics can reshape the gut microbiota structure and promote the production of endogenous beneficial metabolites, indirectly optimizing systemic and local immune environments through immune signal transduction mediated by the gut-nose axis (85). These multifaceted mechanisms provide a solid theoretical basis for postbiotics as an adjunctive treatment for AR.

The application scope of postbiotics has expanded to various immune-related diseases. Specific bioactive proteins (such as p40, p75, and HM0539) have been confirmed to possess significant intestinal immunomodulatory and barrier-protective effects (86). These components drive epithelial cell repair and regulate immune responses by activating signaling pathways such as the epidermal growth factor receptor, providing a molecular foundation for precision treatment with postbiotics. In summary, postbiotic therapy holds significant clinical value and broad application prospects in the immunomodulation of AR.

## 6 Challenges and future prospects

### 6.1 Heterogeneity issues: impact of race and dietary habits on baseline metabolite levels

The levels of gut microbial metabolites are driven by the complex interplay of genetic background, environmental factors, and lifestyle, exhibiting significant heterogeneity. Multiple studies have confirmed that plasma concentrations of gut metabolites, such as trimethylamine N-oxide (TMAO), differ markedly across various ethnic groups. This variation reflects not only genetic diversity but is also closely linked to dietary patterns. For instance, research in multi-ethnic communities has found that while TMAO levels are positively correlated with all-cause and cardiovascular mortality, the strength of this association varies among different racial groups. This suggests that the race-specificity of baseline metabolite levels may influence disease risk assessment and the formulation of intervention strategies (87). Furthermore, as a key environmental factor, dietary habits profoundly regulate metabolite production by reshaping the gut microbiota structure and its metabolic functions. High-fiber dietary patterns, such as plant-based and Mediterranean diets, can significantly promote the production of beneficial metabolites like SCFAs, optimizing microbial diversity and improving host metabolic health (88). In contrast, high-fat Western diets tend to induce dysbiosis, reducing the production of beneficial metabolites and increasing pro-inflammatory factors, thereby exacerbating metabolic disorders and immune abnormalities (89, 90).

Under different racial and dietary backgrounds, baseline differences in metabolites are reflected not only in their types and concentrations but also in the activity of metabolic pathways and their regulatory potency on the immune system. Taking butyrate as an example, its abundance varies significantly among populations with different dietary habits; as a key immunomodulatory molecule, butyrate participates in the pathogenesis of immune diseases such as AR by maintaining intestinal barrier integrity and regulating immune cell activity (91, 92). Therefore, neglecting the diversity of research samples may lead to biased results regarding the relationship between metabolites and immune regulation, limiting the generalizability of clinical applications.

Given these differences, future research should broadly include diverse participant cohorts, integrate multi-dimensional data—including genetic background, dietary habits, and lifestyle—and utilize multi-omics technologies to systematically analyze the variation patterns of gut microbial metabolites and their immunoregulatory mechanisms. This will not only help eliminate selection bias resulting from single-population samples but also reveal the specific action pathways of gut-nose axis metabolites in different groups, providing a scientific basis for developing individualized treatment strategies. Considering the significant influence of race and diet on baseline metabolites, the role of gut metabolites in AR immunomodulation may be population-specific; these variables must be fully weighed when formulating precision intervention protocols (87, 88).

### 6.2 Dose-response effects

Gut microbial metabolites exhibit a significant dose-dependency in modulating the immune response of AR, a characteristic crucial for elucidating their physiological functions and pathological mechanisms. Taking SCFAs as an example, research has found that butyrate at micromolar levels can effectively inhibit the proliferation of tissue-resident ILC2s and the secretion of type 2 cytokines (such as IL-5 and IL-13). This effect is primarily mediated by the inhibition of HDAC activity, which subsequently downregulates the expression of the key transcription factor GATA3 (31). However, when butyrate concentrations exceed a specific threshold (e.g., 1 mmol/L), cell viability begins to be compromised, suggesting that its immunomodulatory effects operate within a narrow and physiologically relevant effective dose window. This dose-response effect indicates that the immune regulation of metabolites is not a simple linear positive correlation; precise dosage control is essential to avoid excessive immunosuppression or potential cytotoxicity.

Beyond SCFAs, secondary bile acids and tryptophan metabolites also demonstrate dose-dependent immunomodulatory features. Secondary bile acids exert gradient regulatory effects on the mucosal immune environment and epithelial function by activating receptors such as FXR and TGR5. Similarly, microbial-derived indole metabolites, acting as ligands for the AhR, play a critical role in maintaining epithelial barrier integrity and balancing the ILC3/ILC2 ratio, with their regulatory efficacy highly dependent on concentration levels. The varying activation intensity of signaling pathways at different doses ultimately determines the nature and extent of the inflammatory response.

Abnormal fluctuations (either excessively high or low) in metabolite concentrations can induce immune imbalance. Research indicates that environmental pollutants (e.g., DEHP) can interfere with gut microbiota structure and metabolite production, thereby disrupting the expression of intestinal epithelial tight junction proteins and the differentiation balance of immune cells, leading to barrier dysfunction and inflammatory responses (93). Conversely, a deficiency in metabolites results in weakened immunosuppressive signals, rendering the system unable to effectively curb excessive type 2 inflammatory responses, which promotes the occurrence and progression of allergic diseases. Therefore, in clinical translation, the safe and effective dose range of metabolites must be strictly defined to ensure immunomodulatory benefits while avoiding side effects.

Defining the effective dose range is of great significance for guiding clinical intervention strategies. For instance, Traditional Chinese Medicine interventions can achieve the goals of inhibiting ILC2 activation and restoring mucosal homeostasis by regulating gut microbial metabolite levels, yet their dose-dependent mechanisms require further clinical validation (31). Furthermore, the heterogeneity of individual gut microbiota composition leads to significant individual differences in metabolite production and host response, suggesting that future precision dosing regimens should be formulated in conjunction with individualized microbiome profiles (94). In summary, systematically elucidating the dose-response effects of gut microbial metabolites not only helps reveal their underlying immunomodulatory mechanisms but also lays a theoretical foundation for developing safe and efficient therapeutic strategies for AR.

### 6.3 Complexity of metabolite interactions

Gut microbial metabolites play a pivotal role in host immune regulation. Complex synergistic or antagonistic effects exist among different metabolites, and these interactions directly dictate the ultimate biological outcome of immunomodulation. Studies have demonstrated that SCFAs, tryptophan metabolites, fatty acid derivatives, and aromatic amino acid metabolites collectively drive immune responses via distinct signaling pathways. For instance, metabolites derived from the plant glycoside plantamajoside, such as hydroxytyrosol and 3-(3-hydroxyphenyl)propionic acid (3-HPP), not only directly alter the abundance of intestinal SCFAs and tryptophan metabolites but may also indirectly participate in immune regulation by modulating the production of these endogenous metabolites (95). Furthermore, the dynamic equilibrium between phenolic substances and pro-inflammatory or anti-inflammatory molecules like lipopolysaccharide is significant in shaping the immune microenvironment of diseases such as AR.

Interactions between metabolites have transcended single metabolic pathways, forming a multi-level, multi-target metabolic regulatory network. For example, aromatic amino acid metabolites generated by the gut microbiota (such as indolepropionic acid and indole-3-carboxaldehyde) can act synergistically with fatty acid metabolites (such as butyrate and propionate) to activate the AhR and GPCRs, respectively, to maintain immune homeostasis (96, 97). Additionally, certain metabolites like phenylacetylglutamine and phenylacetylglycine have been found to influence systemic signaling through interactions with adrenergic receptors, suggesting that metabolites not only mediate local immune responses but also affect systemic immune and metabolic states through complex cross-talk (21). This intricate network provides a wealth of molecular mechanisms for the immunomodulation of the gut-nose axis.

Systematic analysis of metabolic networks and their downstream signaling pathways is a primary focus of current research. Through multi-omics integration and metabolic pathway modeling, researchers can deeply reveal the interactions between metabolites and their precise regulation of immune cell signal transduction. For instance, the natural product ginsenoside Rg3 activates the AhR/MAPK pathway by modulating gut microbiota metabolism to improve immune function, fully exemplifying the potential of metabolic networks in immune intervention (98). Consequently, a profound analysis of the complex interactions among metabolites and their signal transduction mechanisms will facilitate the development of precision therapeutic strategies for immune-related diseases such as AR.

### 6.4 Safety and efficacy assessment in clinical translation

Gut microbial metabolites and their associated therapies have demonstrated immense potential for the immunomodulation of AR, yet evaluating their safety and efficacy during clinical translation is of paramount importance. Currently, evidence regarding the safety of long-term application of such metabolites or probiotic preparations remains relatively limited. Although some clinical studies have not observed significant adverse reactions, they are constrained by small sample sizes and short follow-up periods, which are insufficient for a comprehensive assessment of long-term potential risks (99, 100). Therefore, future research urgently needs to conduct large-scale, long-term clinical trials to establish safety benchmarks by systematically monitoring the incidence of adverse events and their long-term impact on the immune system.

Scientifically rigorous experimental design is the core to ensuring the reliability of efficacy assessments. Current research on probiotics and metabolite interventions for AR exhibits significant heterogeneity, involving differences in strain selection, dosage, administration routes, and treatment duration, which leads to inconsistent conclusions (99, 101). Furthermore, the diversity of participant populations (e.g., age, disease severity, comorbidities) and the lack of standardized outcome measures (e.g., symptom scores, immunological markers) weaken the comparability and generalizability of research findings (1, 102). To this end, future clinical studies should strictly adhere to randomized, double-blind, placebo-controlled principles, define primary and secondary endpoints, adopt standardized efficacy evaluation systems, and integrate multi-omics technologies to deeply analyze mechanisms of action, thereby enhancing the scientific rigor and credibility of the research.

At present, some clinical studies maintain a cautious stance on the actual efficacy of metabolite interventions. For instance, a randomized controlled trial on prenatal prebiotic supplementation for high-risk infants to prevent atopic dermatitis did not achieve significant efficacy, suggesting potential limitations of single-intervention approaches (103). Meanwhile, modified traditional Chinese medicine formulas, such as Yu-Ping-Feng-San variants, have shown good tolerance in improving AR symptoms and quality of life, yet statistical improvements in symptom scores remain non-significant. This implies that multi-target combined interventions may offer greater clinical advantages (102). These results emphasize that individual differences must be fully considered during the clinical translation process, and composite treatment strategies should be explored and scientifically promoted based on robust evidence.

### 6.5 Technical and methodological challenges

Research into the role of gut microbiota and their metabolites in the immunomodulation of AR has driven the widespread application of multi-omics technologies, such as metabolomics and immunomics. However, the integrative analysis of multi-source heterogeneous data still faces severe challenges. First, metabolomics and immunomics data are characterized by high dimensionality and extreme complexity; achieving efficient and precise data fusion is a core difficulty in current research. For instance, although studies using AR mouse models have revealed bidirectional regulatory mechanisms of the gut-nose axis, signal noise and heterogeneity during data integration have limited the depth of result interpretation (3). Consequently, developing specialized joint analysis algorithms and software tools tailored for gut metabolites and immune indicators is key to improving research quality.

Secondly, the lack of standardized detection methods and data interpretation frameworks restricts the comparability of research results and the efficiency of clinical translation. Currently, the detection of gut microbial metabolites primarily relies on mass spectrometry-based techniques. While these allow for quantitative analysis, inconsistencies in sample pretreatment, derivatization protocols, and instrument parameters across different laboratories lead to poor data consistency (104). Similarly, immunomics testing involves a variety of cytokines and immune cell subsets, yet it likewise lacks unified calibration standards and reporting norms. Establishing comprehensive operating guidelines covering the entire process—from sample collection and processing to detection and analysis—has become an urgent need for the development of this field.

Thirdly, the diversity of gut microbial metabolites and their dynamic evolution under different physiological and pathological states place high demands on the precise capture of their spatiotemporal characteristics. The concentrations of key metabolites are significantly influenced by dietary habits, sampling time, and storage conditions. Therefore, enhancing the reliability and biological interpretability of metabolomics data requires optimizing sampling strategies, strictly controlling preservation conditions, and improving detection sensitivity, all in combination with dynamic monitoring technologies.

Furthermore, immunomics technologies such as single-cell RNA sequencing and flow cytometry have shown significant advantages in resolving the heterogeneity of immune cells along the gut-nose axis. However, the high-dimensional data they generate pose severe challenges to computational resources and analysis algorithms. Effectively integrating metabolomics data to construct multi-layered interaction networks between metabolites and immune cell functions remains a technical bottleneck that urgently needs to be broken (25). This requires not only advanced statistical methods but also reliance on artificial intelligence and machine learning technologies to deeply reveal complex molecular regulatory mechanisms.

### 6.6 Construction of personalized diagnosis and treatment strategies

As understanding of the pathogenesis of AR deepens—particularly regarding the immunomodulatory roles of microbiota and their metabolites in the gut-nose axis—constructing personalized diagnosis and treatment strategies has become essential for enhancing clinical efficacy and reducing recurrence rates. First, integrating patient microbiome profiles with metabolite signatures allows for the more precise identification of individual differences in pathological states and immune responses. By leveraging high-throughput sequencing and metabolomics, researchers can identify specific microbial taxa and metabolic biomarkers correlated with AR symptom severity and therapeutic response. This deep analysis based on immunophenotyping not only facilitates a better understanding of a patient’s pathophysiological features but also guides the formulation of individualized interventions, such as targeted dietary adjustments and the application of specific probiotic formulations or metabolic supplements, thereby achieving precision immune regulation (105, 106).

Secondly, multidisciplinary team (MDT) collaboration is core to advancing precision medicine for AR. Integrating the expertise of specialists in otolaryngology, immunology, microbiology, nutrition, and bioinformatics helps achieve a deep fusion of multidimensional data—including clinical phenotypes, molecular diagnostics, and lifestyle factors—to build comprehensive individual health records. For example, the establishment of multicenter real-world registry systems to collect clinical symptoms, treatment responses, and microbiome data allows for the systematic evaluation of the individualized efficacy of Traditional Chinese Medicine and other interventions, demonstrating the significant potential of MDT in clinical translation (107). Furthermore, combining digital health tools with artificial intelligence enables a seamless connection between patient self-management and clinical decision-making, promoting the dynamic optimization and precision closed-loop management of treatment regimens (108).

Finally, personalized strategies must balance the safety and tolerability of immunotherapy. As the only treatment currently capable of altering the natural course of AR, the efficacy of allergen-specific immunotherapy (AIT) is closely linked to a patient’s molecular allergen-specific IgE profile, immune cell status, and microbial metabolic environment. Through precise molecular diagnostics combined with microbiome and metabolic characterization, the populations most likely to benefit from AIT can be screened, and their therapeutic efficacy and potential risks can be predicted, thereby improving treatment adherence and optimizing long-term prognosis (109, 110). Existing research suggests that specific metabolites may assist AIT by enhancing immune tolerance mechanisms, providing a theoretical foundation for individualized combination therapy regimens.

### 6.7 Future research directions

Based on the limitations and challenges of current research, future efforts should focus on advancing the following directions: ①Investigating the role of extracellular vesicles (EVs): Exploring whether EVs produced by gut microbes serve as carriers for metabolites involved in the gut-nose axis; ②Constructing interdisciplinary multi-omics data integration platforms: Establishing open-access databases encompassing the microbiome, metabolome, immunome, and clinical phenotypes, and utilizing big data mining techniques to deeply elucidate the molecular associations within the gut-nose axis; ③Developing technologies for the dynamic monitoring of metabolites: Utilizing cutting-edge technologies such as microfluidic chips and real-time mass spectrometry imaging to achieve precise monitoring of the spatiotemporal dynamics of local metabolites in the gut and nasal cavity; ④Optimizing clinical translation and intervention pathways: Advancing the standardized design of novel therapies such as postbiotics and FMT, and validating their long-term safety and clinical efficacy through real-world studies (RWS); ⑤Carrying out differentiated research across the life cycle: Focusing on the developmental and aging characteristics of the gut microbiota and immune system in children, adolescents, and the elderly, and conducting age-stratified research on the regulatory mechanisms of the gut-nose axis to achieve precision prevention and control for the entire population.

## 7 Conclusion

The gut-nose axis serves as a vital link connecting gut microbial metabolism with nasal mucosal immunity, playing a pivotal regulatory role in the pathogenesis of AR. Intervention strategies based on microbial metabolism—including postbiotic therapy, precision nutritional intervention, and FMT—have demonstrated significant clinical promise. Looking ahead, as research into molecular mechanisms deepens and multi-omics technologies advance, precision interventions based on metabolic networks are poised to drive a paradigm shift in AR treatment, offering new avenues for achieving personalized and etiology-based immunomodulation.
